# Multicharged Zwitterions form Superior Antifouling Interfaces

**DOI:** 10.1002/advs.202514739

**Published:** 2025-10-24

**Authors:** Declan Meehan, Jessica McMaster, Ayantika Kundu, Matthew P. Wylie, Joseph S. Vyle, Karl J. Hale, Marijana Blesic

**Affiliations:** ^1^ School of Chemistry and Chemical Engineering Queen's University Belfast 39–123 Stranmillis Road Belfast Northern Ireland BT9 5AG UK; ^2^ School of Pharmacy Queen's University Belfast 97 Lisburn Road Belfast Northern Ireland BT9 7BL UK

**Keywords:** antifouling, hydration, protein‐resistant, vesicles, zwitterions

## Abstract

The long‐term prevention of unwanted protein and microbial accumulation on surfaces, including medical devices, remains a significant and largely unresolved challenge. Decades of research into antifouling surfaces have suggested that addressing this issue will require a sustained approach focused on incremental advances in chemical design. The creation of highly hydrophilic surfaces has long been recognized as a key strategy, initially pursued through polyethylene glycol‐functionalized coatings. More recently, zwitterionic groups have emerged as effective antifouling moieties. However, the limited chemical diversity of zwitterion‐forming chemical entities has constrained further progress. In this study, an alternative approach to enhancing surface hydrophilicity is presented by employing multicharged zwitterionic molecules (**MZWs**), which increase the density of charged hydrophilic groups per monomer unit. Surfaces functionalized with **MZWs** exhibited 40–45% lower protein adsorption compared to benchmark single zwitterionic molecules. Remarkably, the synthesized **MZWs** spontaneously assemble into vesicular aggregates (130–170 nm) without the need for additives or any form of external force. These findings strongly support further exploration of **MZW**‐functionalization as a novel strategy  to enhance antifouling performance, while remaining readily adaptable via minor modifications to existing synthetic routes used for the incorporation of conventional zwitterions into polymers, self‐assembled monolayers, hydrogels, and nanocarriers.

## Graphical abstract

Bolaamphiphilic multicharged sulfobetaine zwitterion forms self‐assembled monolayer for antifouling applications and vesicular aggregates for drug delivery applications.

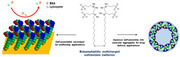

## 1 Introduction

Antifouling surfaces are a critical requirement for many biomedical applications, including medical devices and biosensors.^[^ ^1^ ^]^ They also play a key role in biotechnology,^[^ ^2^ ^]^ in separation and processing, and in industrial food^[^ ^3^, ^4^ ^]^ and marine^[^ ^5^ ^]^ applications. Over the past 50 years, a significant amount of experimental research has gone into understanding the complex mechanisms involved in protein adsorption and in devising materials that can suppress it. Poly(ethylene glycol) (PEG) films have long been recognized as being effective for preventing the non‐specific attachment of proteins and bacteria to solid surfaces.^[^ ^6^ ^]^ Zwitterionic functional materials were first introduced in the early 1980s, as alternatives to PEGs, owing to their superior hydration capacity facilitated by electrostatic interactions and enhanced salt resistance, which render them more suitable for physiological applications.^[^ ^7^ ^]^ Research into surface coatings with integrated zwitterionic functionality has encompassed self‐assembled monolayers (SAMs) that mimic cell membranes, as well as polyelectrolyte multilayer films and polymer brushes. These surface coatings differ significantly in structure, formation, and performance. Zwitterionic polymers with phosphorylcholine functionality were first introduced as antithrombotic materials in the late 1980s by Nakabayashi.^[^ ^8^, ^9^ ^]^ Research in this field intensified noticeably in the early 2000s, when substantial advances were made in the development of protein‐resistant zwitterionic polymer brushes (often incorporating stimuli‐responsive and antibacterial functionalities) by the teams of Jiang^[^ ^10^, ^11^, ^12^, ^13^, ^14^ ^]^ and Zheng.^[^ ^15^ ^]^ In parallel, the development of non‐fouling zwitterionic SAMs flourished, marked by the rich publication output of the Whitesides group.^[^ ^16^, ^17^, ^18^, ^19^, ^20^ ^]^ While SAMs typically contain a single zwitterionic group per monomer, polymer brushes and polymer networks generally achieve a higher degree of hydration due to their greater density of zwitterionic groups along the polymer chains. Polyzwitterionic coatings have been extensively investigated by Lienkamp and co‐workers.^[^ ^21^, ^22^ ^]^

**Figure 1:**
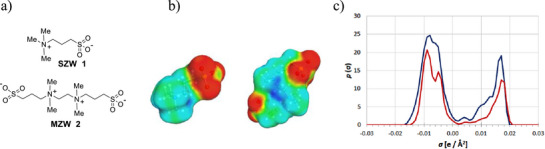
a) Chemical structure of previously synthesized **SZW 1** (upper structure), and **MZW 2** (lower structure). b) Sigma surface of **SZW 1** (left) and **MZW 2** (right), c) Sigma profiles – blue line corresponds to **MZW 2** and red to **SZW 1**.

Following the identification of the most effective zwitterionic groups and the optimization of their density along polymer chains, new strategies to further enhance the hydrophilicity and performance of antifouling coatings began to be explored. In this aspect, our group has previously studied the solvation structure of an aqueous solution of the multicharged zwitterion **MZW 2** (see Figure **1**a) containing two sulfobetaine groups. Analysis by neutron diffraction with isotopic substitution, combined with modeling of the measured structure factors, showed strongly directional local hydration, with 48–52 water molecules shared between **MZW 2** molecules in a first‐shell water network around each zwitterion pair.^[^ ^23^ ^]^ Furthermore, our previous studies provided experimental evidenceof the high kosmotropicity of **MZW** materials, as well as their lack of interaction with charged surfaces, highlighting the potential of **MZWs** to be used in applications where minimization of electrostatic interactions with proteins is required.^[^ ^23^, ^24^, ^25^, ^26^ ^]^

In continuation of this work, our present study demonstrates that **MZW 2**, if used as a functional tail‐group of a monomer, can serve as an effective alternative to conventional zwitterions, offering enhanced hydrophilicity, thus establishing a novel strategy applicable to the design of both SAMs and polymer‐based coatings.

**Scheme 1:**
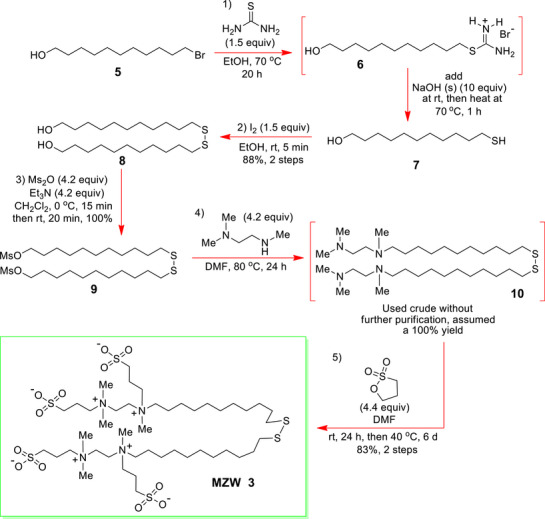
Synthetic route used to access the new **MZW 3**.

Additionally, this study also demonstrates that the novel **MZW** **3** (**Scheme** **1**) can spontaneously form thermodynamically stable vesicular aggregates when dissolved in an aqueous solution, without the need for the application of shear force, external energy, or additives. Spontaneous vesicle formation is a rare phenomenon and has been reported mainly in solutions of double‐chain tetra *n*‐alkylammonium surfactants and mixtures of oppositely charged surfactants with similar chain lengths. These systems do, however, require critical formulation information to be obtained on the suitable molar ratio of oppositely charged surfactants in an aqueous system, to avoid their precipitation,^[^ ^27^ ^]^ and/or the addition of alcohols or co‐surfactants to stabilize the resulting vesicular structures.^[^ ^28^, ^29^, ^30^, ^31^ ^]^ There are reports on vesicle formation by a variety of bolaamphiphiles that are structurally similar to the newly synthesized **3**.^[^ ^32^, ^33^ ^]^ However, the vesiculation process for bolaamphiphiles regularly requires a methanol injection, film formation, extrusion, and sonification,^[^ ^34^, ^35^, ^36^, ^37^ ^]^ or additives such as cholesterol and cholesterol hemisuccinate,^[^ ^38^, ^39^ ^]^ and non‐ionic or ionic surfactants.^[^ ^40^ ^]^ Bolaamphiphiles most commonly form micelles^[^ ^41^, ^42^ ^]^ or nanofibers^[^ ^42^, ^43^ ^]^ in aqueous solution. Reports of the spontaneous formation of vesicular aggregates in unsonicated solutions of bolaamphiphiles without any additive are extremely scarce.^[^ ^44^ ^]^

## 2 Results and Discussion

### 2.1 Modeling

COSMOtherm calculations were performed to evaluate the relative contributions of hydrogen bonding and ion–dipole interactions to the hydration behavior of the previously synthesized **MZW 2** and **SZW** (singly‐charged zwitterion) **1** molecules, which were subsequently employed in this work as functional groups in the monomers **MZW** **3** and **SZW** **4**, respectively.^[^ ^45^, ^46^ ^]^ COSMO‐RS (Conductor‐like Screening Model for Real Solvents) is a theoretical framework that simulates the screening behavior of solutes in solution. It performs quantum chemical COSMO calculations on both solute and solvent molecules to estimate deviations from ideal screening occurring in real solvent systems (see Supporting Information, SI). This method utilizes density functional theory to optimize molecular geometries and calculate electrostatic potentials, generating screening charge densities on the surface of a cavity surrounding the solute that is assumed to be embedded in a conductor‐like medium.^[^ ^47^ ^]^ The result is a 3D polarity distribution (see Figure 1b), which can be condensed into a 2D sigma profile that represents the normalized frequency of surface segments exhibiting a given screening charge density (σ), as shown in Figure 1c. The two peaks in the *σ*‐profile of the **SZW 1** at −0.008 and −0.004 e Å^−2^, correspond to the positive charge located on the nitrogen atom and partially distributed over 11 surrounding acidic hydrogen atoms (note that positive partial charges on atoms cause negative screening charge densities and vice versa). In the same region, but with higher frequency, positive charges were spread in the **MZW 2** molecule. The two peaks with a high screening charge density of +0.016 e Å^−2^ correspond to negatively charged oxygen atoms of the sulfonate group. Since the peaks in the region *σ* >0.01 e Å^−2^ range are associated with the hydrogen bond acceptor ability of the molecule, the *σ*‐profile demonstrates that **MZW 2** molecules have a higher chance of forming hydrogen bonds with water and a higher level of hydration compared with the **SZW 1** described by a lower maximum (Figure 1c).

### 2.2 SAMs

**Scheme 2:**
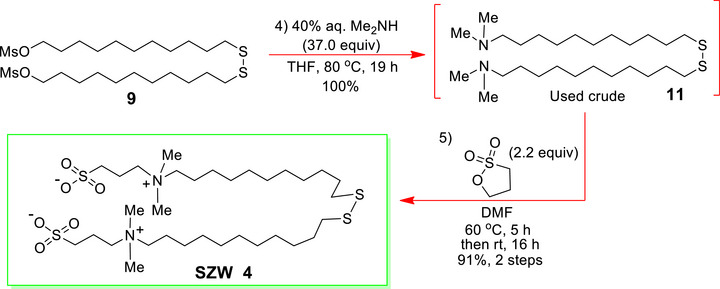
Synthetic route used to access the new **SZW 4**.

Our above findings prompted a comparative study of gold surfaces that had been functionalized with the new long alkyl chain monomers **MZW 3** (**Scheme** 1) and **SZW 4** (**Scheme** **2**). These surfaces were then characterized, and their protein resistance was evaluated. Protein adsorption on SAMs composed of **SZW 4** monomers has been previously investigated, and these monolayers have been established as among the most effective protein‐resistant surfaces.^[^ ^16^ ^]^ Nevertheless, **SZW 4** was synthesized for the present study, and protein adsorption experiments were repeated to ensure a consistent and controlled basis for comparison. Collecting experimental data under uniform conditions was considered essential, as the extent of protein adsorption can vary significantly depending on numerous factors, such as the measurement technique, the protein type and its concentration in solution, surface exposure time, temperature of evaluation, buffer composition (including pH and ionic strength), and the structural quality of the SAM layer.

The synthetic routes, closely aligned with those employed by Zu *et al*.^[^ ^48^ ^]^ and Hjelmeland^[^ ^49^ ^]^ were developed to access the long alkyl chain **MZW 3** and **SZW 4** monomers used to functionalize the gold‐coated substrates. They are shown in **Schemes** 1 and 2 (experimental details provided in the SI).

Given that multiple literature reports have demonstrated that alkanethiols and their corresponding dialkyl disulfide precursors form SAMs with identical structure and similar formation kinetics,^[^ ^50^, ^51^ ^]^ we selected the dialkyl disulfide forms of compounds **MZW** **3** and **SZW** **4** for this study. This approach offers the practical advantage of enabling the long‐term storage of key intermediate **9** in a more stable form.

SAMs are considered convenient entities for the control of surface roughness, free energy, chemical affinity, and interfacial phenomena of great practical importance, such as wettability, lubrication, corrosion, and protein resistance.^[^ ^18^, ^52^, ^53^, ^54^ ^]^ Even though SAMs have been intensively studied for more than five decades,^[^ ^55^ ^]^ particularly those formed by adsorption of alkane thiolates on gold substrates, the effect of monomer tail‐group size, geometry, and charge on the monomer packing density and layer ordering remains incompletely understood.^[^ ^18^ ^]^ The driving force for the formation of SAMs includes the chemisorption of the surface‐active head‐group onto the substrate. Additionally, van der Waals interactions between long alkyl chains and, in the case of charge‐bearing monomers, electrostatic interactions between oppositely charged groups, along with potential hydrogen bonding, further effect monomer packing density.^[^ ^55^ ^]^ In this study, SAMs have been prepared in aqueous solution by the chemisorption of zwitterion‐functionalized dialkyl disulfides on a gold‐coated surface (see SI for experimental details). Chemically, the process is postulated to involve reductive cleavage of the S─S bond in these starting materials and the formation of a thiolate bond to gold(I).

Surface energy is an important macroscopic property of SAMs that can be readily quantified through experimental measurements. It is commonly used to evaluate the hydrophilic or hydrophobic nature of a surface and to correlate these characteristics with other properties such as wettability, lubrication, and protein adhesion.^[^ ^56^ ^]^ The Owens‐Wendt‐Rabel‐Kaelble (OWRK) method is a widely adopted approach for calculating the surface free energy of a solid.^[^ ^4^, ^57^ ^]^ It assumes that the surface free energy can be represented by the sum of two independent components: polar and dispersive. This approach allows for the analysis of the relative contributions of polar and dispersive interactions and could provide an indication of the extent of exposure of hydrophilic groups and nonpolar chains at the interface. Using the OWRK Equation (1) and measuring the contact angle of two liquids with known interfacial tension, γ_*lv*_, with their polar, $\gamma_{lv}^{p}$, and dispersive components, $\gamma_{lv}^{d}$, we were able to determine the polar, $\gamma_{sv}^{p}$, and the dispersive component of surface energy, $\gamma_{sv}^{d}$, for **SZW**‐ and **MZW**‐ functionalized SAMs (see also the SI).

(1) $$\sqrt{\gamma_{sv}^{d}\cdot \;\gamma_{lv}^{d}}+\sqrt{\gamma_{sv}^{p}\cdot \;\gamma_{lv}^{p}}=0.5\;\cdot \gamma_{lv}\cdot \left(1+cos\theta_{\gamma }\right)$$

The results are summarized in **Table** **1**. SAMs prepared from boths **MZW** **3** and **SZW** **4** monomers yielded hydrophilic surfaces that exhibited similar surface free energy and water contact angles of (52.5 ± 1.5)° and (53.5 ± 1.5)°, respectively (commonly, surfaces with water contact angles *θ* > 65° are considered hydrophobic, while those with *θ* < 65° are classified as hydrophilic).^[^ ^58^ ^]^ Furthermore, the polar and dispersive components of surface energy for both zwitterionic SAMs were nearly identical, indicating similarly ordered monolayers with the hydrophilic functional groups oriented toward the interface and a comparable degree of exposure of the non‐polar alkyl chains.

**Table 1:** Comparative data for SAMs and Gibbs Monolayers formed of **SZW 4** and **MZW 3**: polar, $\gamma_{sv}^{p}$, and dispersive, $\gamma_{sv}^{d},$ component of surface free energy, average surface roughness, *R_a_*, frequency shift, *Δf*, on adsorption of lysozyme and bovine serum albumin (BSA) proteins, area per molecule in liquid‐gas monolayer, *a*.

SAM						Gibbs Monolayer
	$\gamma_{sv}^{p}$[mNm^−1^]	$\gamma_{sv}^{d}$[mNm^−1^]	*R_a_* [nm]	*Δf* [Hz],_Lys_	*Δf* [Hz],_BSA_	*a* [nm^2^]
**SZW 4**	29 ± 1	32 ± 1	1.48	32 ± 1	19 ± 1	0.43 ± 0.08
**MZW 3**	28 ± 1	32 ± 1	1.66	19 ± 1	11 ± 1	0.96 ± 0.08

**Figure 2:**
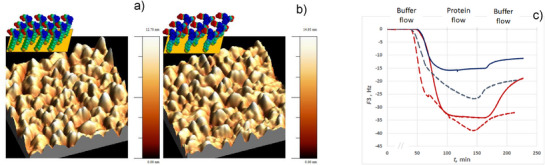
Surface topography of a) **SZW 4** – and b) **MZW 3 –** functionalized SAM; c) frequency drop versus f time; solid lines represent BSA, while dashed lines represent the lysozyme adsorption experiment; red and blue colors represent **SZW 4** – and **MZW 3** – functionalized SAM, respectively.

On the other hand, it is well established that microscopic surface roughness contributes to the apparent hydrophobicity of a surface by increasing the contact angle of a liquid droplet,^[^ ^59^ ^]^ thereby influencing the determined surface energy. We hypothesized that the bulkier **MZW** group could lead to increased surface roughness (see schematic inserts in Figure **2**a,b to visualize the bulkiness of the tail‐groups). To investigate this, atomic force microscopy was employed to assess nanoscale topographic variations. The average surface roughness, expressed as the arithmetic mean of absolute height deviations, was comparable for SAMs composed of **MZW** **3** and **SZW** **4**, as summarized in Table 1. Rather high roughness values of 1.48 and 1.66 nm were recorded for the **SZW** **4** and **MZW** **3** SAMs, respectively. Given that the calculated full molecular lengths in equilibrium gas‐phase geometries of thiols formed from **SZW 4** and **MZW 3** are 1.85 and 2.17 nm, respectively, the observed roughness suggests that the SAMs likely consist of monomeric islands in upright or slightly tilted orientations, surrounded by molecules lying nearly parallel to the surface. This is consistent with previous observations that bulky tail‐groups hinder tight packing, resulting in voids, defects, and increased height variation.^[^ ^51^ ^]^ Low levels of ordering due to steric effects and intermolecular interactions of tail‐groups have been previously reported for SAMs terminated with hydroxyl^[^ ^60^ ^]^ and carboxylic acid groups.^[^ ^61^ ^]^

### 2.3 Protein Adsorption

Following the initial characterization of the SAMs, protein adsorption studies were conducted using two model proteins: lysozyme, a small, positively charged protein at physiological pH, and BSA, a medium‐sized, negatively charged protein. Various techniques, such as Surface Plasmon Resonance,^[^ ^62^ ^]^ Quartz Crystal Microbalance (QCM),^[^ ^63^, ^64^ ^] 125^I‐radiolabeling,^[^ ^65^ ^]^ and Field‐Effect Transistors,^[^ ^66^ ^]^ have been employed in the literature to study protein‐surface interactions. While these techniques usually reveal consistent trends in protein adsorption on a studied surface, they regularly yield differing absolute values of adsorbed protein due to variations in measurement principles. The QCM technique commonly reports a much higher adsorbed mass because it measures the “wet” mass of adsorbed protein with a hydration layer.^[^ ^65^, ^67^ ^]^ In this study, QCM was used to examine the behavior of SAMs functionalized with **SZW 4** and **MZW 3** under continuous flow of phosphate buffer (pH 7.4) and protein solutions (see SI for experimental details). Notably, both **MZW 3** and, to a lesser extent, **SZW 4** functionalized SAMs exhibited a significantly delayed baseline stabilization during the buffer flow, compared to typical stabilization time found for CH_3_‐terminated SAMs or pure gold surfaces, in what was attributed to an extended hydration process at the interface. Extensive surface hydration was also indicated by massive dissipation values with a prolonged frequency decrease. The results of the protein adsorption experiments are presented in Figure 2c, showing a frequency drop between two plateaux, which is proportional to the adsorbed mass over time during the three key stages: buffer flow (conditioning), protein flow (adsorption), and repeated buffer flow (removal of weakly attached protein). A summary of the quantitative data is provided in Table 1. The results clearly demonstrate that **MZW 3**‐functionalised SAMs exhibit superior resistance to protein adsorption achieving an approximately 40–45% reduction in adsorbed mass relative to **SZW** 4 counterparts for both lysozyme and BSA. These results also indicate that surface energy and contact angles are not reliable predictors of a surface's susceptibility to protein adsorption. **MZW 3** was also found to retain its antifouling properties in Dulbecco's phosphate‐buffered saline (DPBS) containing Mg^2^⁺ and Ca^2^⁺, as evidenced by a frequency decrease of (14 ± 1.2) Hz upon lysozyme adsorption (see Figure S10, SI). The reduced extent of protein adsorption compared to that observed in PBS can be partially attributed to the slightly higher ionic strength of DPBS relative to PBS (0.19 vs 0.17 m).^[^ ^16^ ^]^ However, we  are of opinion that the observed reduction also arises from differences in buffer composition. This interpretation is supported by our previous experiments (unpublished data available upon request), which revealed that buffer composition can significantly influence protein adsorption even when the buffers possess identical pH values.

### 2.4 Vesicular Aggregate Formation

**Figure 3:**
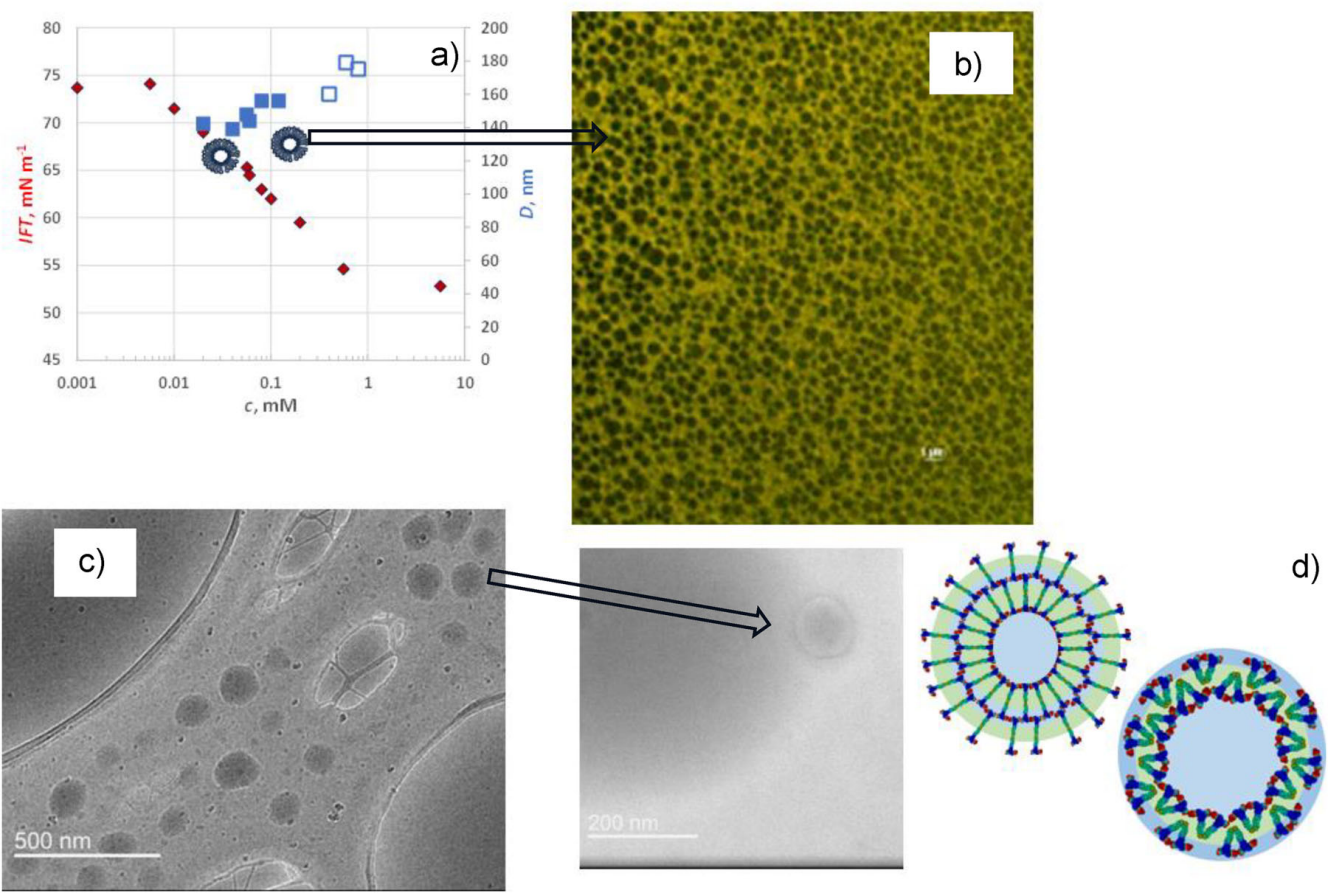
a) Interfacial tension (red diamonds) of aqueous **MZW 3** solutions plotted against its concentration. The secondary Y‐axis represents the hydrodynamic diameter of aggregates, as determined by dynamic light scattering (DLS). Solid blue squares indicate measurements with a polydispersity index (PDI) below 0.3, corresponding to a single, well‐defined population of aggregates. At higher concentrations, empty blue squares denote increased PDI and the emergence of additional aggregate populations, likely indicating the onset of aggregate growth or structural transitions. b) Microscopic image of vesicular aggregates formed in an aqueous solution saturated with dye Nile Red of 0.05 mm **MZW 3**. c). Cryo‐TEM images of vesicular aggregates formed in a 0.05 mm aqueous solution of **MZW 3** (dark‐grey cycles). d) Two hypothetical molecular arrangements within the vesicular aggregates: one featuring extended chains and the other displaying U‐shaped (bent) conformations of **MZW**‐**3** molecules.

To gain insight into the packing behavior of **MZW 3** and **SZW 4** in aqueous environments, we examined their interfacial and solution self‐assembly. In the case of **MZW 3**, several competing effects were anticipated. On one hand, the steric hindrance imposed by the bulky terminal zwitterionic group may hinder the stabilizing effect of van der Waals interactions between neighboring chains. On the other hand, the presence of multiple oppositely charged moieties could enhance attractive electrostatic interactions, thereby promoting cohesive packing and monolayer formation.^[^ ^68^ ^]^ Since **MZW** **3** and **SZW** **4** have identical chain length, the molecular packing in a monolayer should largely reflect the impact of the terminal groups. We measured the surface tension of aqueous solutions containing the **MZW 3** and **SZW 4** as a function of concentration and determined the corresponding area per head group using Gibbs equation^[^ ^69^ ^]^ (see Table 1, Figure **3**a; Figure S8a, SI). The experimentally determined head group areas −0.96 nm^2^ for **MZW 3** and 0.43 nm^2^ for the **SZW 4** can likely be attributed to the differences in the terminal group volumes, with no evidence of additional strong cross‐headgroup interactions in the case of **MZW 3**.

Despite its low interfacial activity with surface tension plateauing at (53 ± 0.5) mNm^−1^ and reduced packing density in Gibbs adsorption monolayers, **MZW 3** exhibits a clear propensity for self‐association in aqueous solution. Driven by the hydrophobic effect and the thermodynamic incentive to minimize free energy, **MZW 3** forms spherical aggregates across a broad concentration range (10^−2^ to 1 mM). DLS analysis revealed that these aggregates possess a relatively uniform hydrodynamic diameter of (150  ±  20) nm and a polydispersity index (PDI) below 0.3 (see Figure 3a; Figure S9, SI). High‐resolution microscopy, combined with encapsulation of the solvatochromic dye Nile Red, confirmed the spherical morphology of the aggregates (Figure 3b). Cryo‐TEM images further supported their size and the expected layered structure (Figure 3c). The aggregates exhibited good colloidal stability, with only an 8% increase in size observed over two weeks. They also demonstrated efficient encapsulation of a hydrophobic solute (see Figure S8b, SI) underscoring their potential as drug carriers and as an alternative to PEGylated nanocarriers, which, unlike zwitterion‐decorated vesicles, are susceptible to accelerated blood clearance upon repeated administration.^[^ ^70^, ^71^ ^]^

Preliminary experiments investigating the effect of ionic strength on aggregate size revealed that vesicular aggregates undergo a transition to spherical micellar aggregates with a diameter of (6 ± 1) nm in 0.17 M PBS, as determined by DLS. Because this size closely corresponds to that of the studied BSA (≈7 nm) a kinetic study on the BSA adsorption on the surface of **MZW 3** aggregates in PBS by DLS showed a single particle population of ≈7 nm throughout a 48 h period, precluding a definitive conclusion regarding the nature of the interaction (experimental details provided in the SI). Nevertheless, the observed ionic‐strength‐induced vesicle‐to‐micelle transition may be advantageous for formulations intended for personal and healthcare applications, where a rapid release of encapsulated vesicular contents is desired upon administration.

## 3 Conclusion

While the long‐term antifouling performance of **MZW 3** in complex biological media remains to be established through further comprehensive studies, our findings provide a strong theoretical and experimental basis for use of multicharged architectures as a promising strategy for the enhancement of antifouling effectiveness of well‐documented single‐charge betaine zwitterionic structures previously implemented in polymeric surfaces,^[^ ^72^, ^73^, ^74^, ^75^, ^76^, ^77^, ^78^ ^]^ self‐assembled monolayers,^[^ ^16^, ^19^ ^]^ nanocarriers,^[^ ^79^, ^80^, ^81^ ^]^ and gels.^[^ ^82^ ^]^ Notably, the **MZW** highly hydrated surface layers may contribute to enhanced surface lubrication – an important factor in reducing friction, infection risk, and complications during medical device insertion. **MZW**‐based bolaamphiphiles were shown to spontaneously aggregate into vesicular and micellar structures. Their advantageous features, including straightforward preparation, scalability, intrinsic antifouling properties, and high aqueous stability, position them as versatile candidates for further exploration in applications that require stealth characteristics and rapid load release in the presence of salts.

## Conflict of Interest

The authors declare no conflict of interest.

## Supporting information



Supporting Information

## Data Availability

The data that support the findings of this study are available in the supplementary material of this article.
