# Supplementary material for: Multicharged Zwitterions form Superior Antifouling Interfaces
Source: Adv Sci (Weinh). 2025 Oct 24;13(3):e14739. doi: 10.1002/advs.202514739 (PMC12806515; doi:10.1002/advs.202514739)
Supplement: Supplementary file 1 — Supporting Information [file ADVS-13-e14739-s001.pdf]

Supplementary Information

Multicharged Zwitterions Form Superior Antifouling Interfaces

Declan Meehan,[a] Jessica McMaster,[a] Ayantika Kundu,[a] Matthew P. Wylie,[b] Joseph S. Vyle, [a] Karl J. Hale,[a] Marijana Blesic[a]*

[a] D. Meehan, J. McMaster, A. Kundu, Dr. J. S. Vyle, Prof. K. J. Hale, Dr. M. Blesic
School of Chemistry and Chemical Engineering
Queen's University Belfast,
39-123 Stranmillis Road, Belfast, BT9 5AG, Northern Ireland, UK
E-mail: m.blesic@qub.ac.uk

[b] Dr. M. P. Wylie
School of Pharmacy
Queen's University Belfast
97 Lisburn Road, Belfast, BT9 7BL Northern Ireland, UK

Modelling: COSMO-RS (conductor-like screening model for real solvents) is a concept developed to address the insufficiencies of the theoretical approach of dielectric continuum solvation models. Rather than considering solvent as a dielectric continuum *i.e.* homogeneously polarisable medium surrounding a solute molecule, COSMO-RS introduces conductor-like screening of a solute. It performs COSMO calculation for both solute and solvent *i.e.* for all molecules present in a system. It considers as a starting point an ideal screening of a polar solute molecule by solvent on their contact points and afterward calculates the deviation from ideal screening occurring in real solvent system (nonideal pairings). Density Functional Theory was used for the optimisation of parameters and the calculation of electrostatic potential and the screening charges produced on a cavity formed around a solute when embedded in a conductor-like solvent. The equilibrium geometry of both SZW **1,** SZW **4**, MZW **2,** and MZW **3** molecules were optimised at the ground state in gas using the ωB97X-D/6-31G* density functional model to generate output in XYZ-geometry-file format using SPARTAN'20 software for quantum chemical calculations. Quantum chemistry program package Turbomole was used to generate COSMO files using the input files created in SPARTAN'20. The 3D structures of zwitterionic compounds with their surface charge distributions, sigma profiles, and sigma potentials were generated using COSMOthermX (software) - BIOVIA COSMOtherm, version 20.

Materials:

Reagents used for syntheses 11-Bromo-1-undecanol (Sigma Aldrich, 98%), Thiourea (Alfa Aesar, 99%), NaOH (Sigma-Aldrich ≥98%), Methanesulfonic anhydride (Sigma Aldrich, 97%), Iodine (Sigma Aldrich, 98.7%), Dimethylamine, 40 wt.% solution in water (Thermo Scientific Chemicals), 1,3-Propane Sultone (Alfa Aesar, 99%), N, N, N'-Trimethylethylenediamine (TCI Chemical, 97%) were used as purchased without further purification. The lysozyme from

chicken egg white and bovine serum albumin (BSA) were purchased from Merck Life Science and used without further purification.

Composition of phosphate buffer (PBS, pH 7.4) used was: 0.137 M NaCl, 0.0027 M KCl, 0.01 M Na_2HPO_4, 0.0018 M KH_2PO_4. Composition of Dulbecco's phosphate-buffered saline (DPBS) was: 0.9011 mM $CaCl_2$, 2.683 mM KCl, 0.4919 mM $MgCl_2 \cdot 6H_2O$, 136.893 mM NaCl, 15.216 mM Na_2HPO_4.

<u>Synthesis of MZW 3</u>

11,11'-Disulfanediyldiundecan-1-ol (8)

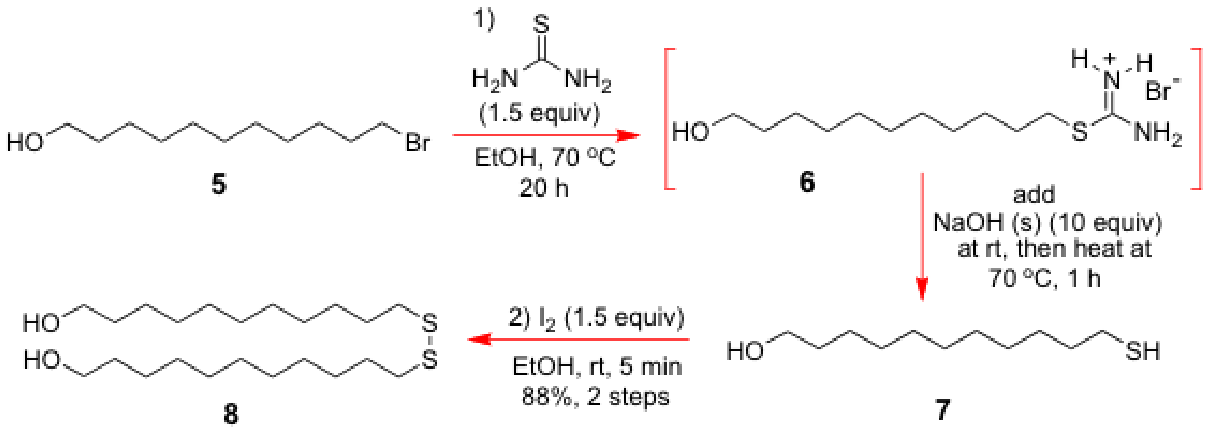

Scheme 1S. Synthetic route used to access **8.**

To a stirred solution of 11-bromo-1-undecanol **5** (10.0 g, 39.8 mmol) in degassed EtOH (200 mL) under Ar was added thiourea (4.55 g, 59.2 mmol, 1.5 equiv.) in one portion and the resulting solution was then heated at 70 °C for 20 h. That solution, now containing **6**, was then cooled to room temperature and solid NaOH (15.92 g, 398.1 mmol, 10.0 equiv.) was added portion-wise with vigorous stirring. The reactants were then heated at 70 °C for 1 h. The solution was again cooled to room temperature, concentrated *in vacuo*, and the crude product (which now corresponded to **7**) was purified by extraction with $CHCl_3$ (600 mL x 3). The organic extract was sequentially washed with H_2O (600 mL x 2) and brine (100 mL x 2). It was then dried over Na_2SO_4, filtered under gravity, and concentrated *in vacuo*. The crude thiol **7** was now dissolved in EtOH (200 mL) and solid I_2 (15.61 g, 59.7 mmol 1.5 equiv.) was added in portions with stirring. When the addition was complete, the reaction mixture was stirred at room temperature for 5 min. The solution was then concentrated *in vacuo*, and the crude residue was extracted with $CHCl_3$ (600 mL x 2). The organic extract was successively washed with 10% aqueous Na_2SO_3 (500 mL) and brine (100 mL x 2). The organic extract was then

dried over Na_2SO_4, filtered under gravity, and concentrated *in vacuo*. The disulfide diol **8** was obtained as a white solid (7.14 g, 17.6 mmol, 88%) and was sufficiently pure to be used directly for the next step. 400 MHz ^{1}H NMR of **8** ($CDCl_3$): δ 3.63 (t, 4H, $HOCH_2$), 2.51 (q, 4H, SCH_2), 1.58 (m, 8H, SCH_2CH_2, and $HOCH_2CH_2$), 1.31 (m, 8H, $S(CH_2)_2CH_2$, and $HO(CH_2)_2CH_2$), 1.28 (m, 20H, $S(CH_2)_3(CH_2)_5$) ppm.

ES+ MS ($C_{22}H_{46}O_2S_2$) m/z = 407.3 $(M + H)^+$, 429.3 $(M + Na)^+$, 813.6 $(2M + H)^+$, 835.6 $(2M + Na)^+$; calc. ($C_{22}H_{46}O_2S_2H$) 407.3018, found 407.3022.

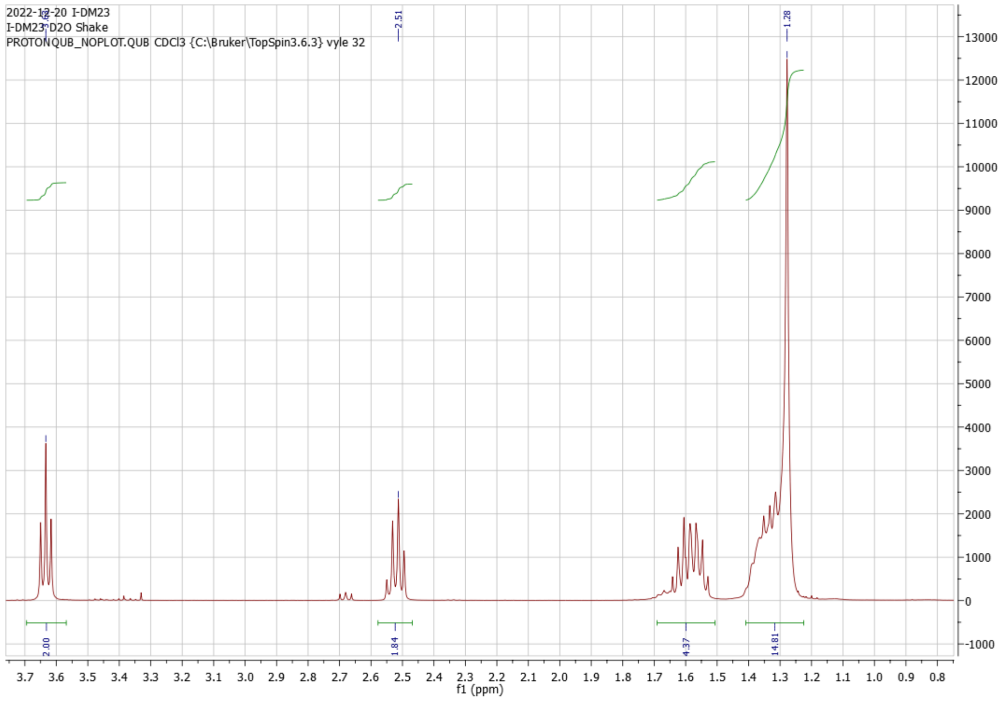

Figure 1S. 400 MHz ^{1}H NMR spectrum of compound **8**.

11,11'-Disulfanediylbis(undecane-11,1-diyl) dimethanesulfonate (9)

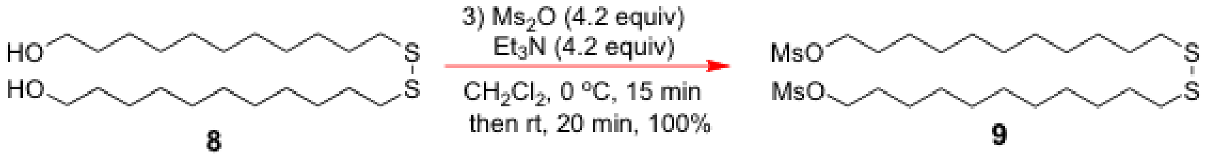

Scheme 2S. Synthetic route used to access **9.**

Diol **8** (6.9 g, 16.9 mmol) was dissolved in dry CH_2Cl_2 (100 mL) and dry Et_3N (9.93 mL, 71.2 mmol, 4.2 equiv.) was added maintaining an Ar atmosphere throughout. The reaction mixture was then cooled to 0 °C, whereupon Ms_2O (12.0 g, 71.2 mmol, 4.2 equiv.) was added portion-wise under argon and the solution stirred for 15 min before being brought to room temperature. The reaction proceeded for 20 min and quenched with $NaHCO_3$ (5% aq. 200 mL) at 0 °C. The product was extracted in DCM (500 mL), H_2O (300 mL x 2), and brine (400 mL). The organics were dried over Na_2SO_4, filtered under gravity, and concentrated under reduced pressure. The product was purified *via* column chromatography, SiO_2, run in pure DCM. The product was obtained as a white solid (9.54 g, 16.9 mmol, 100%).

400 HMz ^{1}H NMR of **9** ($CDCl_3$): d 4.22 (t, 4H, MsO$\underline{CH_2}$), 3.00 (s, 6H, $\underline{CH_3}$ of OMs), 2.68 (t, 4H, S$\underline{CH_2}$), 1.75 (m, 4H, $OCH_2\underline{CH_2}$), 1.67 (m, 4H, $SCH_2\underline{CH_2}$), 1.38-1.28 (m, 28H, $S(CH_2)_2\underline{(CH_2)_7}$) ppm.

ES+ MS ($C_{24}H_{50}O_6S_4$) m/z = 563.3 $(M + H)^+$, 580.3 $(M + NH_4)^+$, 1142.5 $(2M + NH_4)^+$; calc. $(C_{24}H_{50}O_6S_4H)^+$ 563.2569, found 563.2568.

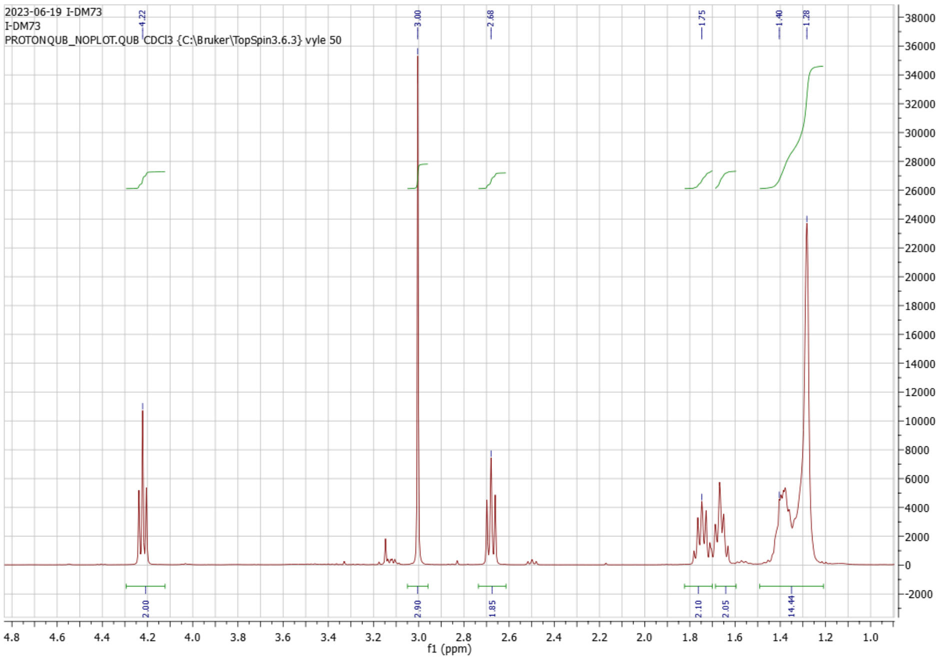

Figure 2S. ^{1}H NMR spectrum of compound **9**.

N^1,$N^{1'}$-(11,11'-Disulfanediylbis(undecane-11,1-diyl))bis(N^1,N^2,N^2-trimethylethane-1,2-diamine) (10)

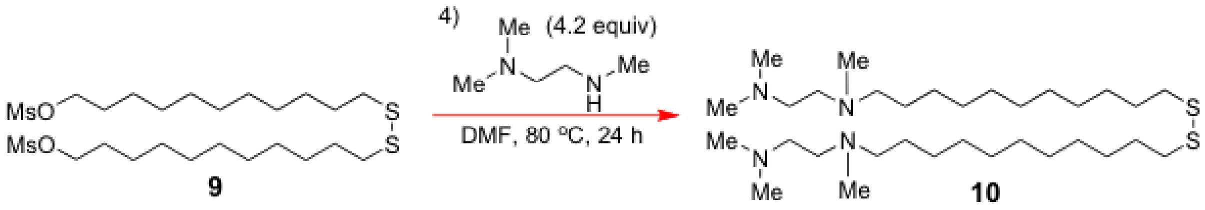

Scheme 3S. Synthetic route used to access **10.**

To a stirred solution of the di-O-mesylate **9** (5.0 g, 8.88 mmol) in dry DMF (13.2 mL) under Ar at 80 °C was added a solution of *N,N,N'*-trimethylethylenediamine (4.83 mL, 37.3 mmol, 4.2 equiv) in dry DMF (3.7 mL) dropwise over 5 min. Heating was continued at 80 °C for 24 h whereafter the reaction mixture was cooled to room temperature and then diluted with CH_2Cl_2 (600 mL). The organic solution was thereafter washed with saturated aqueous Na_2CO_3 (300 mL x 2) and brine (200 mL). The organic layer was dried over Na_2SO_4, filtered under gravity, and concentrated *in vacuo*. The product was obtained as an amber oil and was used directly without further purification for the next step with an assumed 100% yield.

400 MHz ^{1}H NMR ($CDCl_3$) of **10**: δ 2.68 (t, 4H, $S\underline{CH_2}$), 2.43-2.33 (m, 12H, $\underline{CH}N(\underline{CH_2})_2$), 2.25 (m, 18H, $N(\underline{CH_3})_2$), 1.67 (m, 4H, $SCH_2\underline{CH_2}$), 1.46 (m, 4H, $S(CH_2)_2\underline{CH_2}$), 1.37 (m, 4H, $NCH_2\underline{CH_2}$), 1.27 (m, 24H, $S(CH_2)_3(\underline{CH_2})_6$) ppm.

ES+ MS ($C_{32}H_{70}N_4S_2$) m/z = 575.5 $(M + H)^+$; calc. $(C_{32}H_{70}N_4S_2H)^+$ 575.5120 found 575.4547.

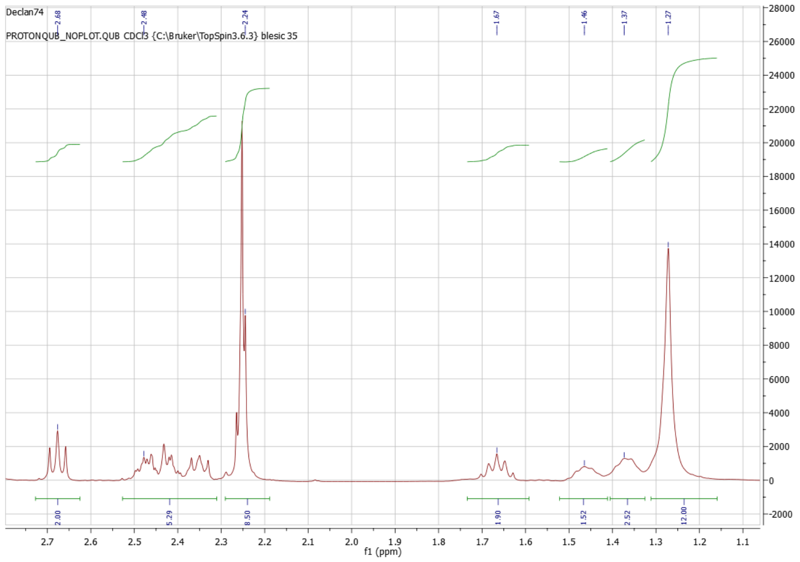

Figure 3S. 400MHz ^{1}H NMR spectrum of tetra-amine-disulfide **10**.

Betaine MZW 3

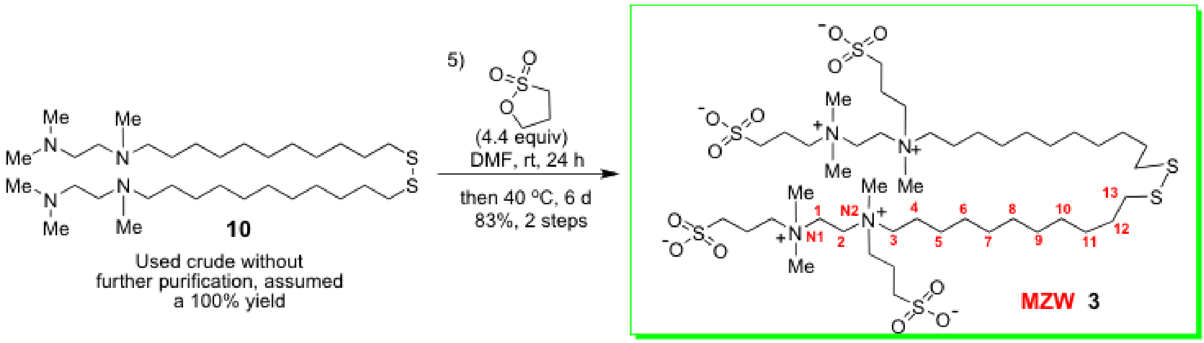

Scheme 4S. Synthetic route used to access **MSW 3.**

To a vigorously stirred solution of a portion of the aforementioned crude tetramine **10** (1.59 g, assumed to be *ca*. 2.77 mmol) in dry MeOH (10 mL) under N_2 was added a solution of 1,3-propane sultone (1.48 g, 12 mmol, 4.4 equiv) dissolved in dry MeOH (5 mL) dropwise over 5 min. The reaction was found to be exothermic and for larger scale preparations initial cooling in an ice bath is recommended. After 24 h of stirring at room temperature, the reaction mixture was subsequently heated at 40 °C for 6 d. The reaction mixture was then concentrated *in vacuo*, suspended in a small volume of acetone, and the resulting solid filtered. It was thereafter washed four more times with minimal quantities of acetone before being transferred to a reaction flask and dried *in vacuo* on a rotary evaporator. It was then dried further under high vacuum. The product **MZW 3** (2.44 g, 83%) was obtained as a colourless-to-very pale yellow, very hygroscopic, soft wax-like material. Thermogravimetric analysis indicated that **MZW 3** remained amorphous likely due to the presence of diastereoisomers in the sample. However a minor endothermic peak was observed at 50 °C that corresponded to a melting event associated with a visible surface crystalline region, which is believed to be formed by the sulfonate group in combination with adsorbed atmospheric moisture. The compound was stored at 4 °C for a period of two years and showed no signs of degradation, as confirmed by ^{1}H NMR analysis.

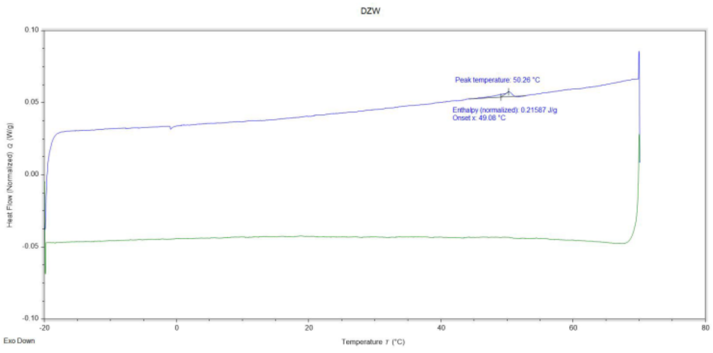

Figure 4S. Thermogravimetric analysis of compound **MZW 3**.

400 MHz ^{1}H NMR spectrum of **MZW 3** (D_2O):

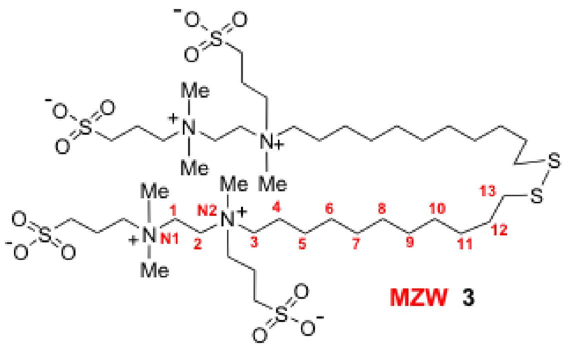

MZW 3

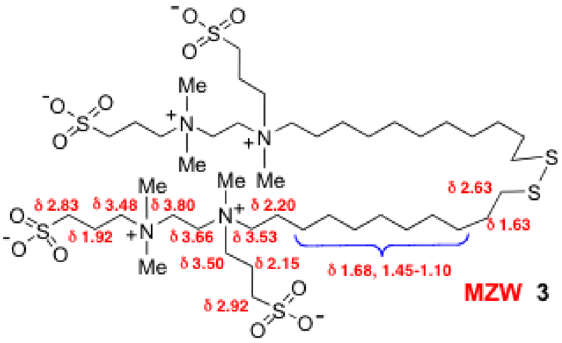

MZW 3

3.80 (br m, 4H, 2 x -(CH_2)- protons that are connected to the **N1**) diastereotopic

3.66 (br m, 4H, 2 x -(CH_2)- protons that are connected to the **N2**) diastereotopic

3.53 ((br m, 4H, 2 x **C(3)**-(C<u>H</u>$_2$)- protons that are connected to the **N2**) diastereotopic

3.50 (br m, 4H, 2 x **N2** -C<u>H</u>$_2CH_2CH_2SO_3^-$); protons diastereotopic due to the chiral **N2** atom

3.48 (t, 4H, 2 x -C<u>H</u>$_2CH_2CH_2SO_3^-$ protons that are connected to the **N1**)

3.28 (s, 12H, 4 x Me groups connected to **N1**)

3.17 (s, 6H, 2 x Me groups connected to **N2**)

2.92 (complex m, 4H, 2 x **N2** -CH_2CH_2C<u>H</u>$_2SO_3^-$)

2.83 (complex m, 4H, 2 x **N1**-CH_2CH_2C<u>H</u>$_2SO_3^-$)

2.63 (br t, 4H, 2 x -(CH_2)- of **C(13)**)

2.20 (br m, 4H, 2 x -(CH_2)- of **C(4)**)

2.15 (complex m, 4H, 2 x **N2** -CH_2C<u>H</u>$_2CH_2SO_3^-$)

1.92 (m, 4H, 2 x -CH_2C<u>H</u>$_2CH_2SO_3^-$ that are connected to the **N1**)

1.63 (br m, 4H, 2 x **C(12)**-C<u>H</u>$_2$-)

1.68 (br) and 1.45-1.10 ((complex br m, 28H, 2 x -(C<u>H</u>$_2$)$_7$- **C(5-11)**).

Elemental analysis: $C_{44}H_{94}N_4S_6O_{12} \cdot 10H_2O$: C 41.6% (41.9); H 9.3% (9.2); N 3.9% (4.4) S 15.8% (15.2).

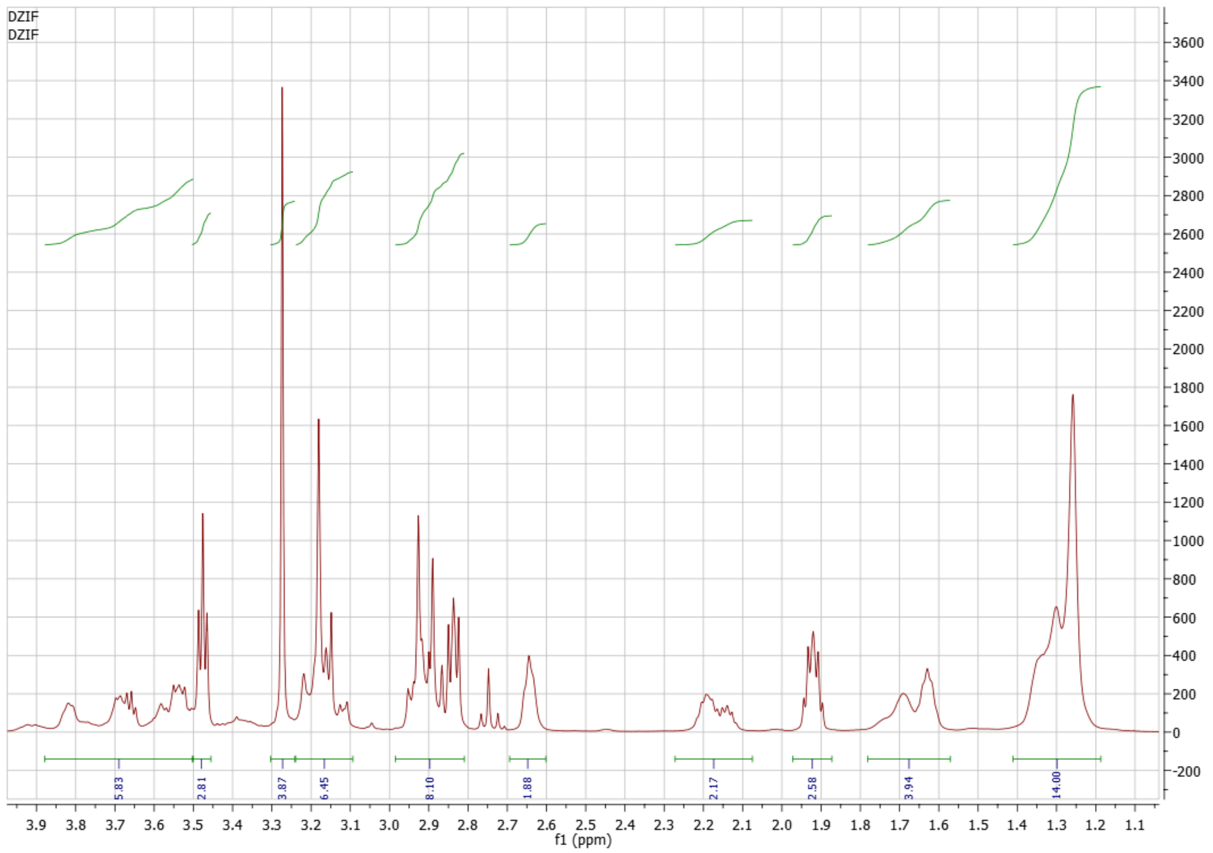

Figure 5S. 400 MHz ^{1}H NMR spectrum of **MSW 3.**

Synthesis of Betaine SZW 4

11,11'-Disulfanediylbis(*N*,*N*-dimethylundecan-1-amine) (11)

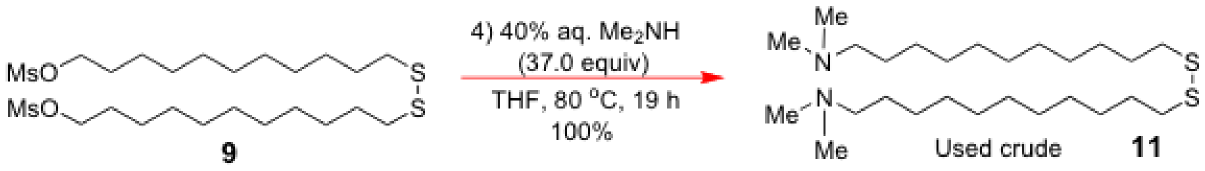

Scheme 5S. Synthetic route used to access **11.**

To a vigorously stirred solution of the di-O-mesylate **9** (4.84 g, 8.60 mmol) in THF (40 mL) under Ar at room temperature was added Me_2NH (40% wt in H_2O, 24 mL, 318 mmol, 37 equiv.). A reflux condenser was fitted to the flask, maintaining the Ar atmosphere, and the reactants were then heated at 80 °C for 19 h. The solution was thereafter concentrated *in vacuo* and the crude residue dissolved in $CHCl_3$ (150 mL x 3) washed successively with saturated aqueous Na_2CO_3 (100 mL) and brine (50 mL). The organic extract was dried over

Na_2SO_4, filtered under gravity, and concentrated *in vacuo*. The crude product was obtained as a brown oil (3.96 g, 8.60 mmol, assumed 100 %) and used directly without further purification.

400 MHz ^{1}H NMR of **11** ($CDCl_3$): δ 2.70 (t, 4H, SCH_2), 2.25 (t, 4H, NCH_2), 2.22 (s, 12H, N$(CH_3)_2$), 1.67 (m, 4H, $SCH_2$$CH_2$), 1.46 (m, 4H, $S(CH_2)_2$$CH_2$), 1.37 (m, 4H, $NCH_2$$CH_2$), 1.27 (m, 24H, $S(CH_2)_3$$(CH_2)_6$) ppm.

ES+ MS ($C_{26}H_{56}N_2S_2$) m/z = 461.4 $(M + H)^+$; calc. $(C_{26}H_{56}N_2S_2H)^+$ 461.3963, found 461.3884.

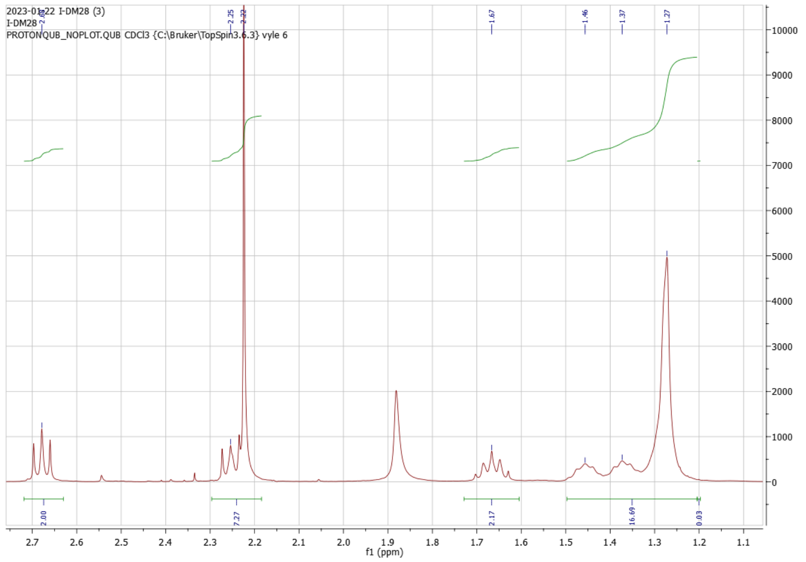

Figure 6S. 400 MHz ^{1}H NMR spectrum of diamine-disulfide **11**.

Betaine SZW 4

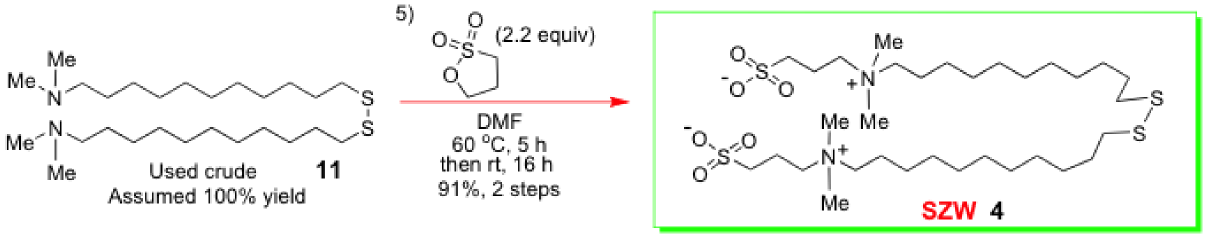

Scheme 6S. Synthetic route used to access **SZW 4.**

To a vigorously stirred solution of the crude diamine **11** (3.96 g, 8.60 mmol) in dry DMF (40 mL) under Ar at 60 °C was added solid 1,3-propane sultone (2.15 g, 18.93 mmol, 2.2 equiv)

in one portion. Stirring was continued for 5 h, whereafter the solution was cooled to room temperature and allowed to stand for 16 h. Anhydrous Et_2O (150 mL) was then added and the solution was cooled to 0 °C for 30 min. A precipitate of **SZW 4** formed, which was collected *via* suction filtration and washed with anhydrous Et_2O (150 mL x 3). The collected solid was dissolved in MeOH (100 mL) and concentrated *in vacuo*. The product **SZW 4** (5.5 g, 7.80 mmol, 91%) was obtained as a burnt orange solid.

400 MHz ^{1}H NMR of **SZW 4** (D_2O): δ 3.40 (m, 4H, $N\underline{CH_2}(CH_2)_2SO_3^-$), 3.24 (m, 4H, $N\underline{CH_2}(CH_2)_{10}$), 3.04 (s, 12H, $N(\underline{CH_3)_2}$), 2.85 (q, 4H, $NCH_2\underline{CH_2}CH_{22}SO_3^-$), 2.66 (t, 4H, $S\underline{CH_2}$), 2.12 (t, 4H, $S\underline{CH_2}$), 1.65 (m, 8H, $SCH_2\underline{CH_2}$, $NCH_2\underline{CH_2}$), 1.26 (m, 28H, $S(CH_2)_2(\underline{CH_2)_7}$) ppm.

ES+ MS ($C_{32}H_{68}N_2O_6S_4$) m/z = 705.4 $(M + H)^+$; calc. $(C_{32}H_{68}N_2O_6S_4 + H)^+$ 705.4039, found 705.4020.

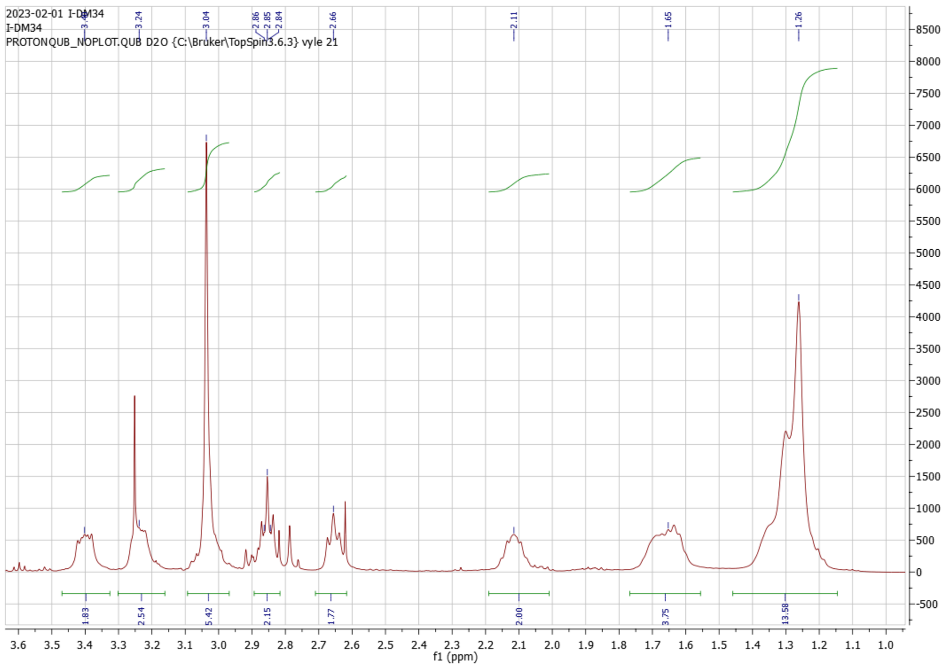

Figure 7S. ^{1}H NMR spectrum of compound **SZW 4**.

Preparation of SAMs: QSense gold-coated disk-shaped sensors functionalised with **MZW 3** or **SZW 4** monomers, forming self-assembled monolayers (SAMs), were used in all experiments. SAMs were prepared by immersing gold-coated QCM sensors in a 1 mM aqueous solution of the zwitterionic monomers for 48 h at room temperature. The sensors

were then thoroughly rinsed with Milli-Q water and ethanol, then dried under a stream of nitrogen prior to adsorption studies.

Atomic Force Microscopy (AFM) measurements were performed using an Oxford Instruments MFP-3D Infinity using the amplitude modulation-frequency modulation (AM-FM) tapping mode. The image analysis was conducted with WSxM software.[1]

Nikon Spinning Disc Super Resolution W1 SORA microscope system was used for to study **MZW 3** or **SZW 4** aggregates in an aqueous saturated solution of Nile Red.

Cryo-TEM Analysis

TEM grid was prepared using an Emitech K550X glow discharge unit for 30 seconds. Sample was loaded on to the TEM grid at a concentration of 0.06 mM (5 μL) and plunge frozen using a Leica EM GP2 in liquid ethane. Sample analysis was carried out using a JEM-21OOPlus equipet with a Gatan OneView camera, a high resolution 16-megapixel CMOS camera, bright field STEM detector, Oxford instruments X-MaxN TLE EDS detector, Gantan Enfinium EELS detector, and HADDF detector. The sample preparation was identical to the one used for dynamic light scattering (DLS) measurements.

Protein Adsorption Study: A Biolin Scientific QSense Initiator was used to study protein adsorption in a flow cell at a controlled temperature of 23°C. The flow rates for the protein and buffer solutions were set to 0.01 mL min^{-1} All protein solutions were freshly prepared prior to the adsorption study at a concentration of 1 mg mL^{-1} in PBS buffer (pH 7.4). The measurements are done in duplicates and the highest found error of ± 1Hz was reported for all measurements.

Repeating the experiment with three-week-old and used SAM-coated gold sensors produced the same protein adsorption as fresh samples, indicating a good stability of the coating on the sensor.

An example calculation of the reduction in hydrated protein mass adsorbed on **MZW 3** SAMs relative to **SZW 4** SAMs is presented in the Table 1S below. The frequency change was determined based on the difference between the plateau region at the end of the conditioning step immediately prior to protein injection and the value recorded at the end of the rinsing process. Since the layers formed on the QCM sensor were highly hydrated, as evidenced by

the substantial increase in dissipation, the observed frequency change could not be converted directly into the “dry” mass of protein using the Sauerbrey equation, which is invalid under these conditions.

Table 1S. Example calculation of the reduction in BSA mass adsorbed on **MZW 3** SAMs relative to **SZW 4** SAMs.

Experim. No.	protein	$\Delta f_{SZW\ 4}$, Hz	$\Delta f_{MZW\ 3}$, Hz	% of Relative Reduction of adsorption
1.	BSA	20.53	11.26	(20.53-11.26)/20.53 x100 = 45%
2. (duplicate)	BSA	18.58	11.22	(18.58-11.22)/18.58x100=40%

Surface Tension and Static Contact Angle Measurements: Surface tension of aqueous solutions was measured with the drop shape analysis method, using a calibrated DSA25 instrument from Krüss GmbH equipped with Krüss ADVANCE software. Droplets of the relevant solution were suspended from a blunt-tipped needle and the surface tension reported as an average of at least 10 measurements. For each individual measurement the dimensions of the drop were captured and fitted using the Young-Laplace equation. Measurements were conducted at room temperature and ambient relative humidity without explicit control. From surface tension data, assuming for these low concentration regimes a monolayer structure at the surface, the minimum area per molecule was calculated, *a*, using the well-known Gibbs equation.[2]

The contact angle measurement was carried out according to the protocol described in literature.[3] A droplet of 0.5 µl volume is formed at the tip of a needle and gently lowered onto the surface. As the needle is raised, the droplet detaches and advances across the surface. The average of five consecutive measurements is reported. Milli-Q water and Diiodomethane were used for determination of surface energy of SAMs.

Fluorescence measurements were performed using an FS5 Spectrofluorometer (Edinburgh Instruments) to analyse the solvatochromic dye Nile Red encapsulated within samples of **SZW 4** and **MZW 3**. A stock solution of Nile Red was prepared at a concentration of 1.5×10^{-7} M. Solutions containing varying concentrations of **SZW 4** and **MZW 3** were added to the Nile Red solution and allowed to equilibrate for 24 hours prior to measurement. The emission intensity of encapsulated Nile Red at 606 nm was recorded over a range of concentrations (from 0.001 to 10 mM) of **SZW 4** and **MZW 3** (see Figure 8S b)).

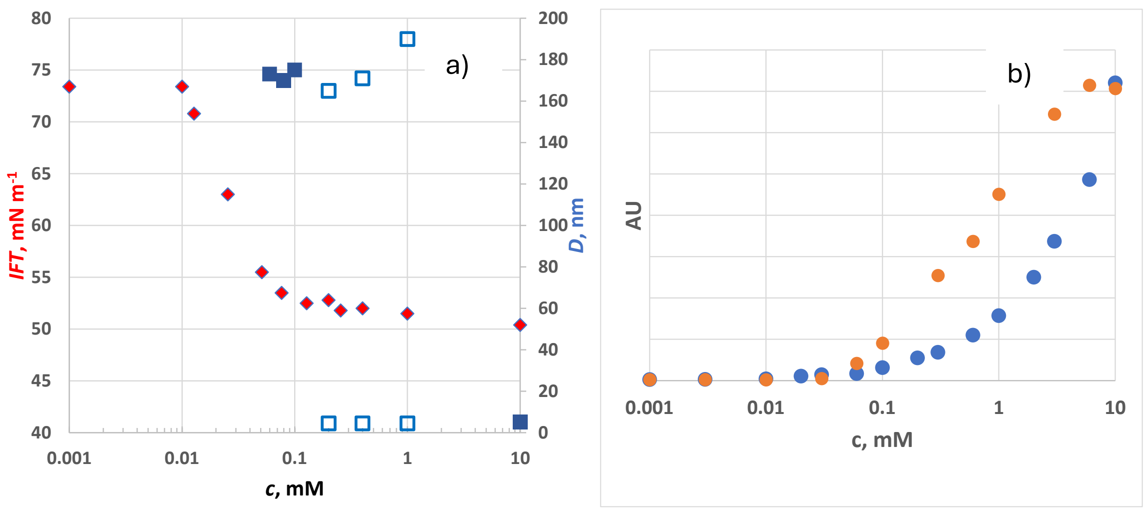

Figure 8S. a) Surface tension *versus* concentration curves for **SZW 4** monomer red diamonds. Solid blue squares indicate hydrodynamic diameters measured by DLC at concentrations where a single population of aggregates was observed, with a polydispersity index (PDI) below 0.30. Hollow blue squares represent DLS measurements under conditions where either two distinct aggregate populations were detected or the PDI exceeded 0.35, suggesting the presence of a heterogeneous mixture of aggregates with varying geometries in solution. b) Fluorescence study of encapsulated hydrophobic molecule of Nile Red in **SZW 4** (orange circle) and **MZW 3** (blue circle) as function of concentration.

DLS Measurement: Samples for DLS measurements were prepared by dissolving **MZW 3** or **SZW 4** in Milli-Q water or PBS, both pre-filtered through 0.45 μm microfilters, to obtain stock solutions. Lower-concentration samples were prepared by serial dilution of the stock solutions. All solutions upon dilution were gently mixed by hand, filtered again through 0.22 μm microfilters, and allowed to stand undisturbed for 24h to facilitate aggregate formation.

DLS measurements were performed using a Malvern Zetasizer Nano Series ZS instrument operating at 25 °C with a 633 nm laser diode and a backscattering detection angle of 173°. For each concentration, the average of 3 – 5 measurements was used to determine the intensity-weighted hydrodynamic diameter (sphere-equivalent) and the polydispersity index.

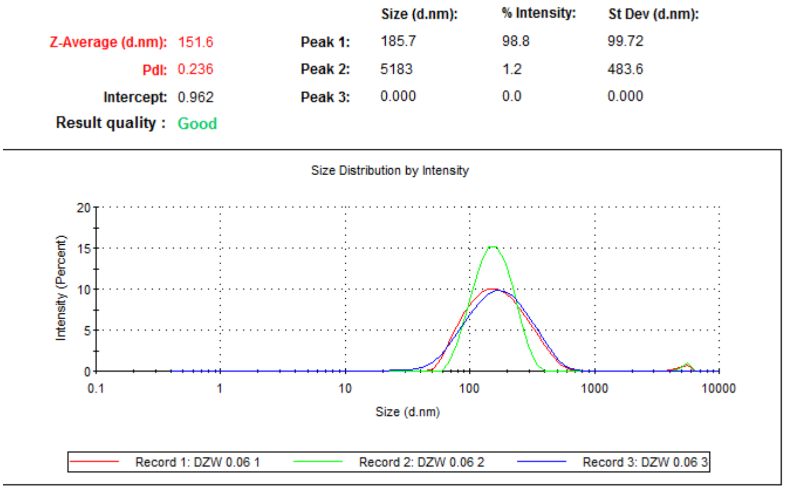

Figure 9S. A sample of DLS measurement of hydrodynamic radius of particles in solution.

To evaluate the interaction between **MZW 3** aggregates and BSA, 0.5 mL of either buffer solution (control) or BSA solution (7 mg·mL^{-1}, prepared in the same buffer) was added to 3 mL of **MZW 3** solutions at concentrations of 0.06, 0.08, 0.10, and 0.12 mM. DLS measurements were performed 30 min, 3 h and 24 h of incubation. All experiments were conducted in duplicate. DLS results indicated the presence of dispersed particles (PDI 0.3-0.5) with an average hydrodynamic radius of (6 ± 1) nm for all samples.

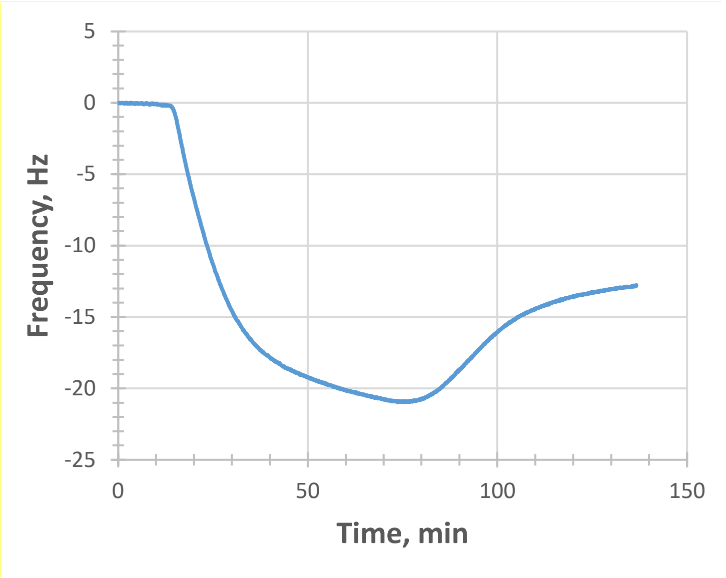

Figure 10S: Frequency drop *versus* time for the lysozyme adsorption experiment on **MZW 3** - functionalised SAM in DPBS buffer.

REFERENCES:

[1] I. Horcas, R. Fernández, J. M. Gómez-Rodríguez, J. Colchero, J. Gómez-Herrero, A. M. Baro, *Review of Scientific Instruments* **2007**, *78*.

[2] M. J. Rosen, *Surfactant and Interfacial Phenomena*, Wiley-Interscience, John Wiley & Sons, New Jersey.

[3] C. D. Bain, E. B. Troughton, Y. T. Tao, J. Evall, G. M. Whitesides, R. G. Nuzzo, *Journal of the American Chemical Society* **1989**, *111*, 321-335.
